# Adaptive multi‐degree‐of‐freedom in situ bioprinting robot for hair‐follicle‐inclusive skin repair: A preliminary study conducted in mice

**DOI:** 10.1002/btm2.10303

**Published:** 2022-02-28

**Authors:** Wenxiang Zhao, Haiyan Chen, Yi Zhang, Dezhi Zhou, Lun Liang, Boxun Liu, Tao Xu

**Affiliations:** ^1^ Biomanufacturing and Rapid Forming Technology Key Laboratory of Beijing, Department of Mechanical Engineering Tsinghua University Beijing People's Republic of China; ^2^ The National and Local Joint Engineering Laboratory of Animal Peptide Drug Development College of Life Sciences, Hunan Normal University Changsha Hunan People's Republic of China; ^3^ Tsinghua Shenzhen International Graduate School, Tsinghua University Shenzhen People's Republic of China; ^4^ East China Institute of Digital Medical Engineering Shangrao People's Republic of China

**Keywords:** automated bioprinting, hair follicle regeneration, in situ bioprinting, skin repair, tissue engineering

## Abstract

Skin acts as an essential barrier, protecting organisms from their environment. For skin trauma caused by accidental injuries, rapid healing, personalization, and functionality are vital requirements in clinical, which are the bottlenecks hindering the translation of skin repair from benchside to bedside. Herein, we described a novel design and a proof‐of‐concept demonstration of an adaptive bioprinting robot to proceed rapid in situ bioprinting on a full‐thickness excisional wound in mice. The three‐dimensional (3D) scanning and closed‐loop visual system integrated in the robot and the multi‐degree‐of‐freedom mechanism provide immediate, precise, and complete wound coverage through stereotactic bioprinting, which hits the key requirements of rapid‐healing and personalization in skin repair. Combined with the robot, epidermal stem cells and skin‐derived precursors isolated from neonatal mice mixed with Matrigel were directly printed into the injured area to replicate the skin structure. Excisional wounds after bioprinting showed complete wound healing and functional skin tissue regeneration that closely resembling native skin, including epidermis, dermis, blood vessels, hair follicles and sebaceous glands etc. This study provides an effective strategy for skin repair through the combination of the novel robot and a bioactive bioink, and has a promising clinical translational potential for further applications.

## 1 INTRODUCTION

The skin is composed of the epidermis, dermis, and subcutaneous tissue, and it is the largest and most complex organ in the human body.^1^ It not only serves as a barrier between the human body and the environment, but also performs physiological functions involving the immune and endocrine systems, physiological metabolism, and nerve conduction.^2^ Large area skin defects caused by burns and other accidental injuries or ulcer diseases are a common occurrence, and can lead to fluid loss, water electrolyte disorders, hypoalbuminemia, or severe infection.^3^, ^4^ However, the self‐repairing ability of skin is limited and wounds larger than 4 cm in diameter do not heal well without intervention,^5^ leading to numerous studies seeking to develop improved skin wound repair techniques.

The requirements for skin wound repair are unique, including (i) rapid healing, (ii) personalization, and (iii) functionality. The first point relates to the requirement for skin wounds to be treated rapidly following injury to prevent fluid loss or infection. Second, the irregular nature of skin wounds, which vary in morphological characteristics and location, necessitates personalized treatment. Finally, the regenerated skin must have appendant organs that support normal physiological functions.

Current skin wound repair methods include autologous skin transplantation, artificial skin substitutes, and three‐dimensional (3D) bioprinting.^6^, ^7^, ^8^, ^9^ Among these, covering the excised full‐thickness wound with autologous skin grafts is considered the “gold standard” for treating severe wounds because skin grafts offer versatility and the capacity for self‐regeneration. However, the applicability of autologous skin grafts remains restricted by the limited supply of available donor sites,^10^ making it difficult to reconstruct the skin damaged by large wounds. Artificial skin substitutes can act as wound coverage and be used as a space filler to create a vital protective layer in cases of severe tissue loss and facilitate the recovery of damaged tissues. Nevertheless, owing to the lack of necessary skin appendages such as hair follicles, the newly formed skin lacks the functionality of normal skin. In addition, the standardized production of artificial skin substitutes means that patient‐specific requirements cannot be satisfied.^11^ The 3D bioprinting technique has the capacity to deliver bioink to specific target sites on a layer‐by‐layer basis to construct tissues or organs capable of performing biological functions and activities. As such, it has been applied in numerous applications.^12^ A new branch of bioprinting, in situ bioprinting, was proposed in 2007, and refers to the direct printing of bioink at a defect site to create or repair living tissues.^13^ Since its inception, in situ bioprinting has shown great progress in the repair of superficial and internal tissues.^14^, ^15^, ^16^ According to previous studies,^17^, ^18^ existing setups for in situ bioprinting are typically based on an open‐loop three‐axis motion platform, which implies that the printer can only perform calibrate‐then‐print operations and is only applicable to static target surfaces. Recently, Zhu et al. described a closed‐loop system to perform in situ 3D printing on a moving hand and deformable lung to compensate the movement of the printed target,^19^, ^20^ but this compensation system was based on a three‐degree‐of‐freedom (three‐DoF) printer which cannot satisfy the stereotactic bioprinting of skin repair due to the lack of DoF Furthermore, no bioprinting approach can yet fully replicate the morphological, biochemical and physiological properties of native skin.^17^, ^21^, ^22^, ^23^, ^24^, ^25^ Most of them constructed 3D‐bioprinted skin through vividly mimic the layered structure consisted of epidermis and dermis but ignored the necessary appendages important for physiological functions, and the incorporation of additional cell types and the patterning of more representative extracellular matrix (ECM) components is necessary.

**FIGURE 1:**
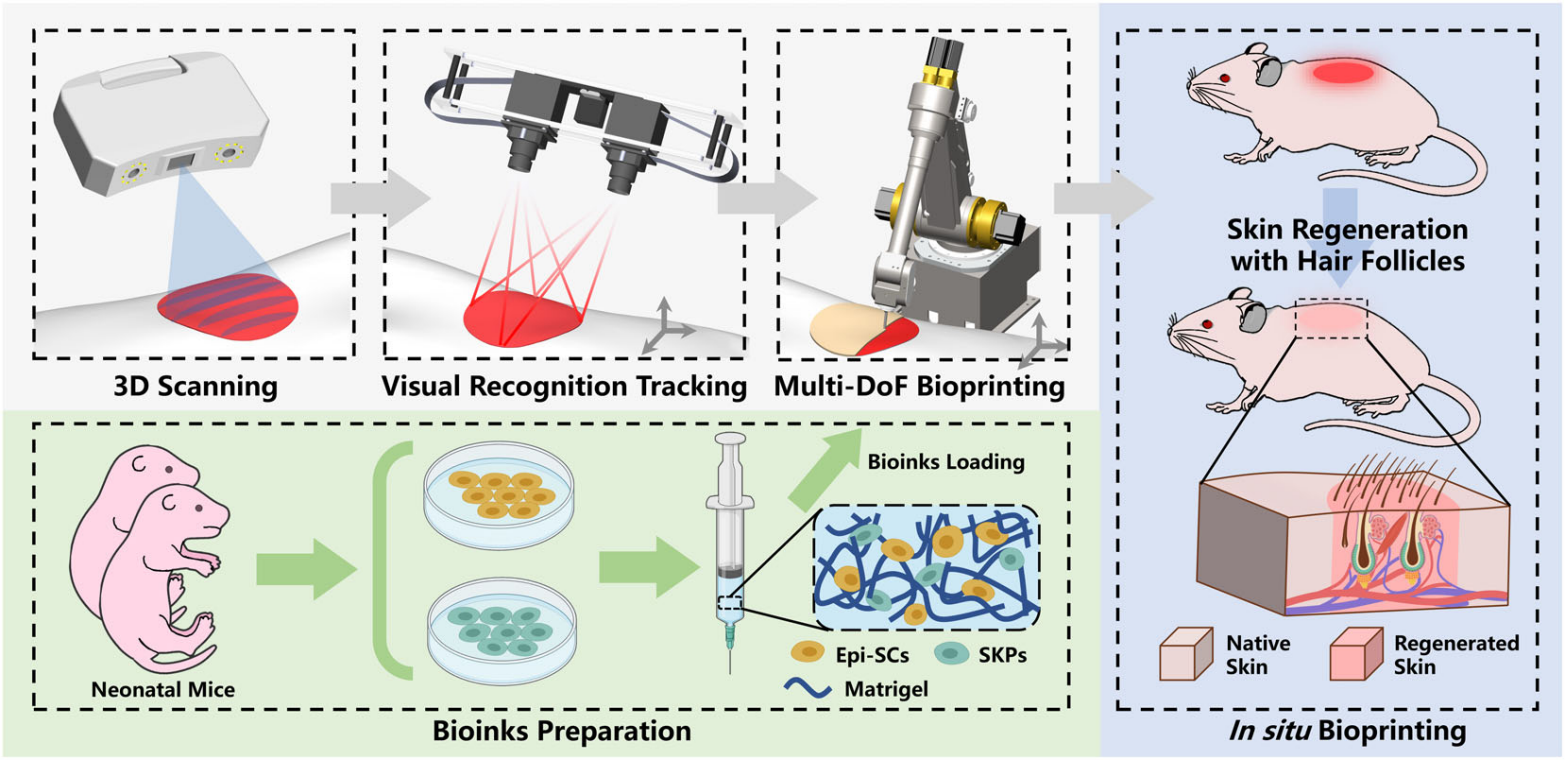
Complete workflow schematic of the adaptive multi‐degree‐of‐freedom in situ bioprinting robot for hair‐follicle‐inclusive skin repair conducted in mice

Herein, we describe the development of an adaptive multi‐DoF in situ bioprinting robot comprising a scanning system, a binocular visual system, and a six‐DoF manipulator to process in situ bioprinting with epidermal stem cells (Epi‐SCs), skin‐derived precursors (SKPs) extracted from neonatal mice and Matrigel as bioink for skin repair. The conceptual design for the method is illustrated in Figure 1. For the needs of personalization, the topographical information of the skin wound was acquired via a structured light scanner to plan the printing path. Wound positions were identified through a binocular camera and fed back to the motion controller of the manipulator, which constituted a closed‐loop feedback system for real‐time wound tracking in order to avoid printing errors due to unintended movement. The six‐DoF manipulator was utilized for prompt cure and rapid healing, except for its portability, the three additional DoF ensured that the print head adjust the printing direction adaptively according to the topography of the skin wounds, facilitating its application to complicated surfaces. Epi‐SCs, as well as dermal stem cells mixed with Matrigel, are used as bioink to pursue functionality requirement owing to their excellent capacity for promoting skin repair and the formation of skin appendages. The aim of the proposed robot is to establish an autonomous workflow for functional skin wound repair that requires minimal human intervention. Our approach advances conventional bioprinting in four aspects. First, integrated 3D scanning is able to acquire quantizable morphology of skin wounds, and adjust printing strategy based on actual situation, thereby achieving personalized skin treatment. Second, the proposed visual system can accommodate six‐DoF wound motion to realize adaptive in situ bioprinting. Third, the manipulator increases the workspace and the ability to print on sophisticated surfaces, which enhances the adaptability for skin repair. Finally, the bioactive bioink confers physiological functionality to the regenerated skin. An in vivo study was conducted in mice to verify the practicability of the proposed bioprinting robot for the repair of functional skin, and to evaluate its application potential in clinical environments.

## 2 MATERIALS AND METHODS

### 2.1 3D scanning and conformal path planning

A structured‐light 3D scanner (Thunk3D) with submillimeter‐level accuracy and resolution was used to acquire the point cloud data of the skin wounds. The point cloud data were then processed using Geomagic Studio software, and the deficient skin model can be acquired through Boolean operations. To fill the skin wounds, the volume of each wound was calculated in order to allocate the bioink appropriately. For small wounds, bioink was delivered directly via a point‐to‐point approach, whereas for large wounds, a planar toolpath was designed using Cura3D software (Ultimaker) and projected onto the reconstructed model to generate the conformal toolpath, which was divided into *i* points as a preset path to control the manipulator, with each point containing information in six coordinates. In addition to the three spatial coordinates, the other three coordinates denote the Euler angle of the point to guide the manipulator as it moves along the normal direction of the toolpath during in situ bioprinting.

### 2.2 Spatial coordinate calculation based on binocular visual identification

Visual identification was performed using a color filter to account for the distinct coloration of skin wounds. Owing to reasons such as glare and shadow, among others, the range of pixel intensity values may vary for different images. A linear normalization procedure was performed to eliminate these effects. During segmentation, the images captured by the binocular camera were converted from the RGB to the HSV color space, making it possible to filter specific colors by adjusting hue, saturation, and value (i.e., brightness). A Gaussian blurring filter with a standard deviation of *σ* = 0.5 was used for image denoising, allowing the image gradients to be obtained before unwanted margins were removed via thresholding.^26^ After applying the final morphological erosion operation, clear wound contours were obtained, and the corresponding barycentric coordinates were calculated. A single camera can obtain only two space object coordinates, which is insufficient for accurate object location. For each frame captured by the camera, the relationship between the wound coordinates in the world coordinate system [*X*_*W*_, *Y*_*W*_, *Z*_*W*_]^T^ and the image coordinate system [*u*, *v*, 1]^T^ can be expressed as

(1) $$\;Z_{o}∙\left[\begin{array}{c} u\\ v\\ 1 \end{array}\right]=H_{c}^{o}∙\left[\begin{array}{c} \begin{array}{c} X_{o}\\ Y_{o}\\ Z_{o} \end{array}\\ 1 \end{array}\right]=K∙P∙\left[\begin{array}{c} \begin{array}{c} X_{o}\\ Y_{o}\\ Z_{o} \end{array}\\ 1 \end{array}\right]=\left[\begin{array}{ccc} f_{x} & s & x_{0}\\ 0 & f_{y} & y_{0}\\ 0 & 0 & 1 \end{array}\right]\left[\begin{array}{cc} R_{3\times 3} & t_{3\times 1}\\ 0 & 1 \end{array}\right]\left[\begin{array}{c} \begin{array}{c} X_{o}\\ Y_{o}\\ Z_{o} \end{array}\\ 1 \end{array}\right]$$

where *K* is the intrinsic matrix of the camera and *P* is the extrinsic matrix. The former matrix denotes the fundamental parameters of the camera; for instance, *f*_x_ and *f*_y_ represent the focal length in different directions, while *x*_0_, *y*_0_, and *s* reflect the distortion. The extrinsic matrix mainly denotes the transformation relation between the camera and world coordinate systems.

Equation (1) obtained from the two frames of the binocular camera are created simultaneously, allowing the 3D coordinates of the skin wound to be acquired. To evaluate the accuracy of the static identification, dots with a diameter of 4 mm were arranged into a 10 × 8 matrix with a pitch of 20 mm, which was used as the recognition target. The lattice coordinates identified by the camera are compared with the real lattice coordinates and used to calculate the recognition error, thereby accounting for the camera being installed in different positions with variable distances and depression angles.

### 2.3 Animals required

Six‐week‐old C57BL/B6 mice (female/male) and five‐week‐old BALB/c nu/nu mice were purchased from Guangdong Medical Laboratory Animal Center. The animals were maintained in a temperature‐controlled environment (20 ± 1°C) with free access to food and water throughout the experiments. This study, including all the procedures involving animals that it entailed, were approved by the Animal Ethics Committee of the Tsinghua Shenzhen International Graduate School.

### 2.4 Isolation and culture of Epi‐SCs and SKPs

Neonatal mouse dorsal skin was harvested from the C57BL/B6 mice approximately 1–3 days after birth. The tissues were cut into sections measuring approximately 2–3 mm^2^ and washed for three times in Hanks' Balanced Salt Solution, then digested with 0.35% dispase II (Sigma‐Aldrich) for 60 min at 37°C. The epidermis was removed from the tissue manually. Epithelial stem cells were isolated for their high adhesive properties.^27^ Briefly, the epithelial layer was cut into slurry and treated with 0.2% collagenase I (Sigma‐Aldrich) for 70 min at 37°C while being agitated, then filtered through a cell strainer with 40‐μm mesh size. The cells were seeded in tissue culture dishes coated with 50 μg/ml collagen I (Sigma‐Aldrich) and incubated in defined keratinocyte‐serum‐free medium epidermal keratinocyte medium (Gibco) with supplements provided by the manufacturer for 60 min. Cells adherent to petri dishes were maintained, while suspended cells were removed through medium change. After reaching 80% confluence, the cells were harvested with Accutase (Gibco) and passaged. SKPs were prepared as previously described.^28^, ^29^ The dermis was treated with 0.3% collagenase I for approximately 60–70 min at 37°C and filtered through a 40‐μm cell strainer. The dissociated cells were incubated in a 10‐cm non‐treated culture dish in the SKP growth medium, which consisted of Dulbecco's Modified Eagle's Medium/F12 in a 3:1 ratio with B27 supplement (both from Gibco), 20 ng/ml of epidermal growth factor (EGF, Peprotech), and 40 ng/ml of basal fibroblast growth factor (bFGF, Peprotech). The incubation was conducted at 37°C in a 5% CO_2_ tissue culture incubator. The SKPs were passaged after digestion with TrypLE™ Express enzyme (Gibco).

### 2.5 Evaluation of in situ bioprinting conducted in mice

The BALB/c nu/nu mice (4–5 weeks old) were anesthetized with sodium pentobarbital (50 mg/kg). Symmetrical full‐thickness skin wounds were created on the back of the mice using the skin biopsy punch with diameter of 2/5/10 mm, as described previously.^30^ The robot was placed near the surgical area to perform pretreatment of bioprinting, including 3D scanning and path planning. Quantitative bioink was transferred to a syringe with an appropriate volume of Matrigel containing 5 × 10^7^ Epi‐SCs per ml and 1 × 10^8^ SKPs per ml. During the printing process, the closed‐loop visual system performed real‐time compensation based on the preset path, which reduce the influence of unintended factors on the printing structures. After bioprinting treatment, skin wounds were then covered with the Tegaderm (3M) transparent dressings and self‐adhering elastic bandages. Four weeks later, the mice were sacrificed, and the numbers of hair shafts were counted under a dissecting microscope and the regenerated tissue samples were harvested for histological analysis.

### 2.6 Alkaline phosphatase staining

The SKPs were harvested and attached to a microslide by centrifugation at 1500 rpm for 5 min. The cells were washed twice with phosphate‐buffered saline (PBS), and fixed with 4% paraformaldehyde (PFA) for 10 min at room temperature (RT). Then the samples were developed with 5‐bromo‐4‐chloro‐3‐indolylphosphate in conjunction with nitro blue tetrazolium (Beyotime Biotechnology) for 4 h in the dark at RT. After washing with PBS, the stained samples were observed under a phase‐contrast microscope (Nikon, Eclipse Ci‐S).

### 2.7 Immunofluorescence staining

Freshly regenerated skin samples were obtained from mice, and fixed in 4% PFA for 8 h. After that, the skin samples were dehydrated in 10%, 20%, and 30% sucrose gradients for 8 h and embedded in tissue freezing medium (SAKURA Tissue‐Tek® OCT Compound). Besides, the SKPs samples were fixed in 4% PFA for 30 min and underwent dehydration followed the above sucrose gradients for 30 min. Frozen tissue sections of the skin tissues and SKPs samples were incubated with different primary antibodies at 4°C overnight, namely, anti‐keratin 14 (1:100, 906004, Biolegend), anti‐nestin (1:100, ab11306, Abcam), anti‐bmp6 (1:100, ab155963, Abcam), anti‐fibronectin (1:100, GTX112794, GeneTex), CD49f‐biotin (1:150, BioLegend), K1 (1:100, 905,601, BioLegend), anti‐CD31 (1:30, g = GTX54379, GeneTex), and anti‐biotin (1:100, 20Raj1, eBioscience). Next, the samples was detected with a TRITC/Cy3 or FITC‐conjugated secondary antibody. Cell nucleus were stained with 4,6‐diamidino‐2‐phenylin‐dole and all the samples were observed under a confocal microscope (FV1000; Olympus).^31^

### 2.8 Hematoxylin and eosin staining

Freshly regenerated skin samples were obtained from mice, and then fixed in 10% formalin or other fixatives for 12–24 h at RT. After dehydration, the tissues were embedded in paraffin for tissue section, and the section slices were rehydrated with 100% ethanol, 95% ethanol, 75% ethanol, and deionized water for 3 min each. Hematoxylin and eosin (H&E) were used to stain the nucleus and cytoplasm respectively, and the prepared samples were observed using a phase‐contrast microscope (Nikon, Eclipse Ci‐S).^32^

### 2.9 Cell viability of bioprinted constructs

Cell viability assay was performed immediately after bioprinting on days 0 (60 min after bioprinting process), 3, and 7 according to the instructions provided by the manufacturer to evaluate the effect of the mechanical disturbance of the nozzle on cell viability. The cells were harvested and suspended in the culture medium, and 0.4% trypan blue dye solution was added at a ratio of 1:10 on the specific day. 20 μl of cell suspension was added to Countstar chamber slide and the cell viability was calculated in Countstar (Countstar Rigel S2) automatically.

### 2.10 Cell proliferation

On culture days 0 (60 min after bioprinting process), 3, and 7, printed constructs were incubated with a mixture of culture medium and cell counting kit‐8 (CCK‐8) (Yeasen) at a volume ratio of 10:1 to investigate cell proliferation. After 1 h incubation at 37°C, the absorbance of formazan dye was determined at 450 nm using a Microplate Reader (Thermo Fisher Scientific). The obtained data was normalized to the cell number according to the pre‐established standard curve. Three samples were tested in each time point.

### 2.11 Flow cytometry analysis

The Epi‐SCs were analyzed through flow cytometry, as described previously.^33^ Briefly, the cells were suspended in PBS containing 1% bovine serum albumin at a density of 10^6^ cells/ml. Cell aliquots (100 μl) were incubated with different fluorescence‐conjugated monoclonal antibodies, namely anti‐CD29‐FITC (1:25, 102205, Biolegend) and anti‐CD49f‐PE (1:25, 313611, Biolegend), or the control isotype IgG at 4°C for 30 min. Flow cytometry (CytoFLEX, Beckman) was used to analyze 10,000 events using Cell Quest software.

### 2.12 RNA isolation and real‐time polymerase chain reaction

Total RNA was extracted using TRIzol (Takara) in accordance with the instructions provided by the manufacturer. First‐strand cDNA was prepared using the PrimerScript™ RT Reagent Kit with gDNA Eraser (Takara) and oligo(dT) primers, and then stored at −20°C. Real‐time polymerase chain reactions (PCR) were performed using SYBR® Green on an analytik Jena qTOWER 3G system. As an internal control, the levels of glyceraldehyde‐3‐phosphate dehydrogenase (GAPDH) were quantified in parallel with the target genes. Normalization and fold changes were calculated using the ∆∆Ct method. The primers used for murine gene amplification were shown in Table 1.

**TABLE 1:** The primers used for murine gene amplification

Genes	Forward	Reverse
*GAPDH*	CGGAGTCAACGGATTTGGTCGTAT	AGCCTTCTCCATGGTGGTGAAGAC
*Nanog*	TCTTCCTGGTCCCCACAGTTT	GCAAGAATAGTTCTCGGGATGAA
*Oct4*	CACCATCTGTCGCTTCGAGG	AGGGTCTCCGATTTGCATATCT
*c‐Myc*	ATGCCCCTCAACGTGAACTTC	CGCAACATAGGATGGAGAGCA
*Sox2*	TCCATGGGCTCTGTGGTCAAG	TGATCATGTCCCGGAGGTCC
*Fibronectin*	ATGTGGACCCCTCCTGATAGT	GCCCAGTGATTTCAGCAAAGG
*a‐SMA*	TGAGCAACTTGGACAGCAACA	CTTCTTCCGGGGCTCCTTATC
*Bmp4*	CAGGGAACCGGGCTTGAG	CTGGGATGCTGCTGAGGTTG
*Collagen I*	GCTCCTCTTAGGGGCCACT	CCACGTCTCACCATTGGGG
*Nestin*	GGTTCCCAAAGAGGTGTCCG	CAGCAAACCCATCAGACTCCC
*PDGFa*	ACGCATGCGGGTGGACTC	GATACCCGGAGCGTGTCAGTTAC
*Akp2*	TCGGAACAACCTGACTGACCC	CTGCTTGGCCTTACCCTCATG

### 2.13 Statistical analysis

All experiments were repeated at least three times, with the results expressed as the mean ± standard deviation (SD) unless stated otherwise. Statistical comparisons between two groups were evaluated using the Student's t‐test. Statistical significance was set at *p* < 0.05.

## 3 RESULTS

### 3.1 3D scanning and real‐time visual tracking

**FIGURE 2:**
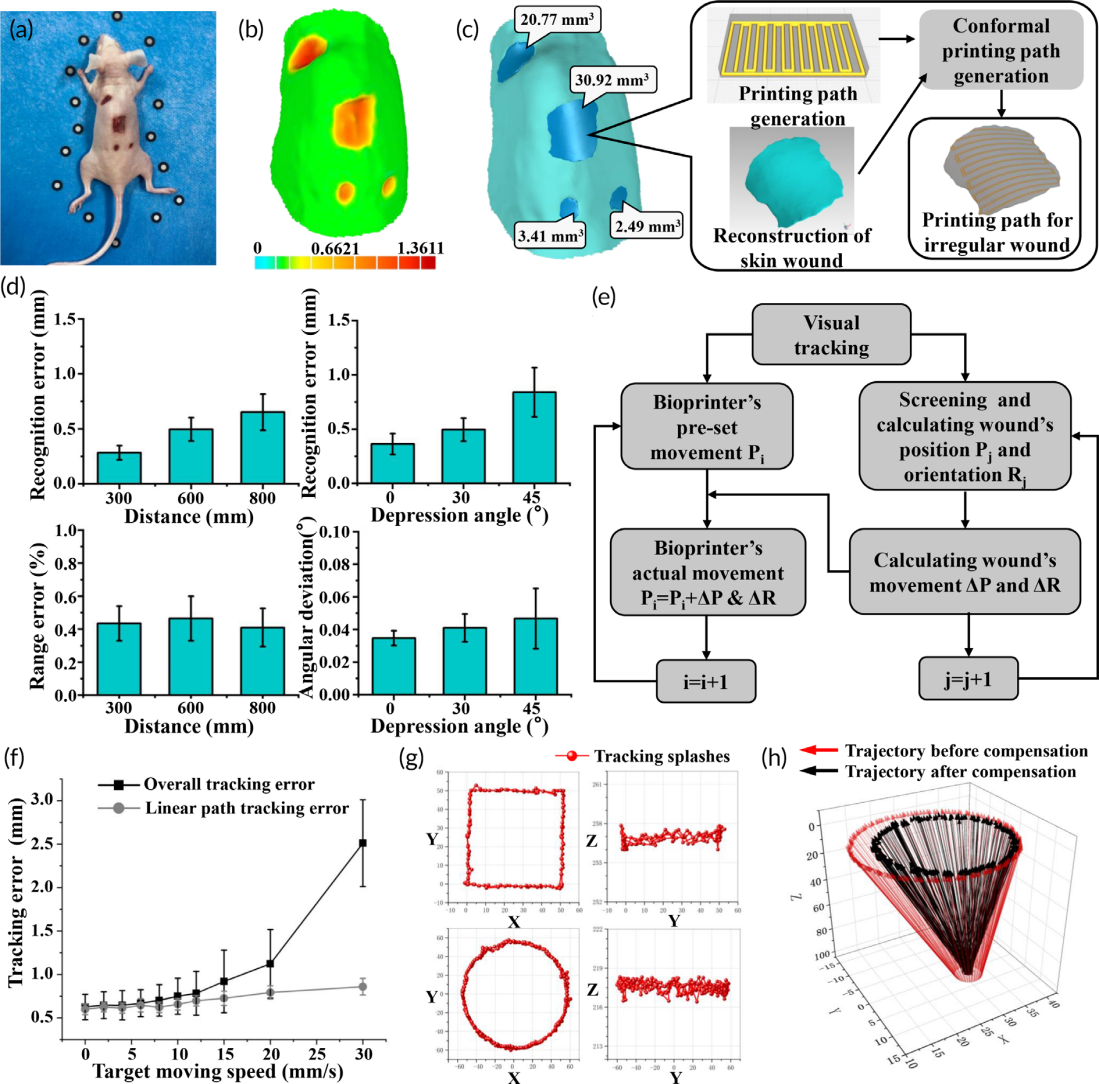
The evaluation of 3D scanning and visual tracking. (a–c) Representative images and results of 3D scanning and conformal path planning. (d) Evaluation of visual recognition for a static target. (e) Algorithm flow chart of the visual tracking. (f) Tracking error for various moving speeds of the target in visual tracking. (g) Tracking splashes trajectory of square path and circular path (top view and side view). Target moves at a speed of 15 mm/s. (h) Tracking trajectory of a fixed point on a rotating shaker

Adaptive bioprinting begins with the assessment of skin wounds, which involves scanning and positioning. First, differently shaped wounds were created on different parts of the selected mouse's back and fiducial markers were set nearby to improve robustness and accuracy for tracking textureless surfaces, which are typical characteristics of skin wounds (Figure 2a). A high‐fidelity skin wound model was acquired via a structured‐light 3D scanner according to Section 2, and the topographical characteristics can be quantified, as represented by the color bar (Figure 2b; Movie S1). In this case, three smaller wounds were treated through the point‐to‐point bioink delivery in strict accordance with the wound volume, and the largest wound (30.92 mm^3^ in volume) was printed according to the regenerated conformal toolpath (Figure 2c). Most skin wounds have clear and distinguished boundaries, with their color differing significantly from native skin, which makes it possible to perform color‐based segmentation of the images for visual tracking. The visual recognition accuracy is reflected by the recognition error, which is defined as the distance between the real position and the calculated position of the recognized target. The results showed that the recognition error varies with the change of distance and depression angle, which can be due to the fixed focal length as well as lens distortion (Figure 2d); however, the errors were always less than 0.8 mm for all experiment groups. To evaluate the overall deviation, including the elimination of the influence of the internal parameters of the camera, the relative range error, *Ed*, and angular deviation, *Ea*, are defined as follows:

(2) $$\;{\mathrm{Ed}}_{i}=\frac{l_{i}-l_{i0}}{l_{i0}},\;{\mathrm{Ea}}_{i}=\tan^{-1}\frac{{\mathrm{dP}}_{i}∙\sin\theta }{l_{i0}},$$

where *l*_*i*_ represents the calculated distance between the object and the camera and *l*_*0i*_ denotes the real distance. Furthermore, *dP*_*i*_ denotes the recognized error for the object and *θ* represents the depression angle.

After eliminating the intrinsic parameters, the range errors were basically consistent for each position and angular deviations were affected by the depression angle to a substantially reduced extent, which indicates that our visual recognition algorithm can satisfy wound identification requirements with high accuracy.

In a rare study involving visual‐based bioprinting,^19^, ^20^ researchers mostly chose an eye‐to‐hand framework, which means that the camera observes the robot within its workspace.^34^ The eye‐to‐hand architecture is a relatively simple and robust framework, but suffers from several problems: (i) camera installation may interfere with the robot's workspace; (ii) movement of the manipulator may block the camera during tracking; and (iii) an appropriate trade‐off between the workspace and tracking accuracies cannot be achieved.^35^ In this study, an eye‐in‐hand structure was used, whereby the camera was mounted in a fixed position on the robot's end‐effector. This is the first time that this structure was applied in robot‐assisted in situ bioprinting. This modified eye‐in‐hand configuration provides an effective solution to the aforementioned shortcomings and raises the possibility of performing adaptive in situ bioprinting inside the human body, because it is impractical to install a fixed camera within the body near the printing device.

In the eye‐in‐hand configuration, the manipulator was controlled by the camera through solving the relation between the camera coordinate system and the end‐effector coordinate system. Here, the eye‐in‐hand system obeys the following identity relation:

(3) Hi∙Hce=Hgw∙Hi,cgew

where Hiew represents the homogeneous transformation matrix of the end‐effector coordinate system relative to the base coordinate system of the manipulator. In addition, Hce denotes the homogeneous transformation matrix of the camera coordinate system relative to the end‐effector coordinate system, which is the solution of Equation (3). Next, Hgw is the homogeneous transformation matrix of the calibration grid coordinate system relative to the base coordinate system of the manipulator, which is constant. Finally, Hicg is the homogeneous transformation matrix of the camera coordinate system relative to the calibration grid coordinate system. The subscript *i* varies and represents different samples.

The identity relation is solved for Hce through multiple sampling and it facilitates visual servoing tracking with the eye‐in‐hand structure. When the binocular camera detects unexpected movement of skin wounds, it automatically compensates for this disturbance to the preset path for bioprinting, thereby ensuring high accuracy. The image acquisition and manipulator control cycles are synchronized, which improves the visual tracking response speed (Figure 2e). To characterize the tracking performance, the robot was instructed to track a given trajectory at different speeds (Figure 2f). This test revealed that the tracking error can be controlled with a precision within 1 mm, even during rapid movement (20 mm/s). Furthermore, the primary source of error appears to occur in the corner of the trajectory (Figure 2g); without this source, the error could be decreased to 0.8 mm even at speeds of 30 mm/s. Skin wounds were considered as rigid bodies with six‐DoF for translation and rotation. The plane of the wounds was determined by identifying *n* points (*n* ≥ 3), allowing the position and orientation of wounds to be captured. The recognition target was placed on a shaker for visual tracking, with the shaker following a circular motion path with constantly changing direction. This is illustrated in Figure 2h, where the movement before and after the compensation are indicated by the red and black arrows, respectively, proving that direction tracking is maintained during rotation.

### 3.2 Control and evaluation of bioprinting on complicated surfaces

**FIGURE 3:**
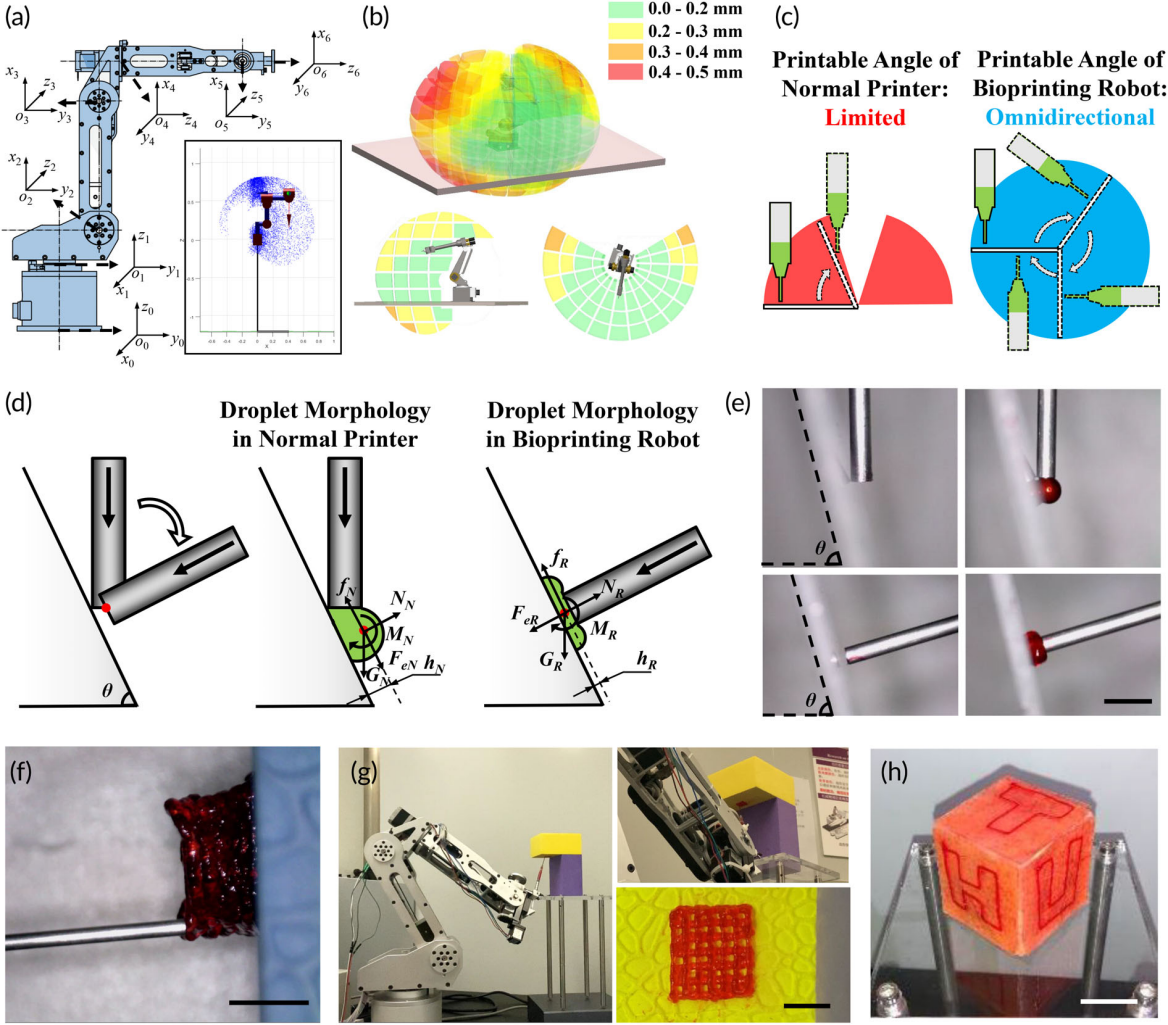
Control and evaluation of bioprinting on complex surfaces. (a) The coordinate system fixed on each joint of the bioprinting robot obtained through D–H method. The bottom right corner denotes a side view of the robot's workspace obtained by Monte Carlo simulation. (b) The workspace of the bioprinting robot and the distribution of the kinematic error involved (axonometric drawing, side view and top view). (c) The printable angle of normal printers (Red) and robotic printers (Blue) on inclined surface. (d) Theoretical analysis of printing on the inclined plane of normal printers and robotic printers from the droplet morphology. (e) Instantaneous microphotographs of droplet morphology between different bioprinters (scale bar: 2 mm). (f and g) Multilayer constructs printed on vertical and inverted surface, respectively by the robotic bioprinter (scale bar: 4 mm for f and 10 mm for g). (h) The letters “THU” printed on porcine skin with different orientations continuously by the robotic bioprinter (scale bar: 2 cm)

The bioprinting manipulator uses a series mechanism with six‐DoF, and its kinetic model can be established using the Denavit–Hartenberg (D–H) method.^36^ The coordinate systems were set at each joint of the manipulator (Figure 3a), with the corresponding transformation relationship expressed as

(3) Ti=cosθi−sinθicosαisinθicosαicosθisinθisinαiaicosθi−sinαicosθiaisinθi0sinαi00cosαidi01i−1

where *a*_*i*_ represents the length of the link, *α*_*i*_ is the link twist, *d*_*i*_ denotes the link offset, and *θ*_*i*_ represents the joint angle. The D–H parameters of the actual bioprinting manipulator are presented in Table S1.

The kinematic model of the manipulator can be obtained by multiplying the transformation matrix of the six joints in turn to realize manipulator control:

(4) T60=∏i=16Tii−1=P4×4=R3×3p3×101,

where T60 represents the transformation from joint 0 to joint 6 and *P* denotes the position of the end of the manipulator, with *R* and *p* representing the orientation and spatial coordinates of the end‐effector, respectively.

The workspace of the manipulator was computed by mechanism analysis and the Monte Carlo method separately in MATLAB 2018 (MathWorks).^37^ Error accumulation is an inherent defect in serial manipulator,^38^ rendering it necessary to evaluate the kinematic error. The manipulator moves inside the entire workspace with a 5 mm pitch and the end‐effector was tracked by a laser to calculate the kinematic error, which was represented in the workspace (Figure 3b). The green section denotes the manipulator with the highest accuracy in the entire workspace (<0.2 mm). A relatively large error occurs at the edge of the workspace; nonetheless, the kinematic error remains <0.4 mm in 93.4% of the workspace. Evaluating the spatial distribution of the kinematic error is helpful for allocating the manipulator appropriately. In the actual bioprinting process, it is possible to optimize the appropriate location of the manipulator to obtain the most accurate workspace feasible for the target repair area.

Typically, the print heads of conventional three‐axis bioprinters are fixed and angled vertically downward, which is an error‐prone configuration when working with complex surfaces such as skin. When the inclination angle of the printing plane increases, the printed threads will struggle to adhere, leading to rolling and slipping. This will affect the fidelity of in situ bioprinting. The adaptability of normal printing and robot‐assisted multi‐DoF printing was evaluated at different inclination angles, revealing that thread skidding of threads occurred when inclination angle increased to 70° in 3‐asix printer. On the contrary, in the robot‐assisted bioprinting, results in a printable angle of 360°, thereby realizing omnidirectional in situ printing (Figure 3c).

Assuming that the center of the print head of the conventional bioprinting and robot‐assisted bioprinting are at the same distance from the plane, in the transient state, the droplets in the two conditions have different microscopic morphologies. Previous studies have shown that friction between hydrogels and the printing plane can occur via a stick‐slip phenomenon, which is related to the thickness, contact area, and driving force of the hydrogels.^39^, ^40^ In normal printers, droplets are approximately spherical, leading to the centroid *h* being far from the plane. A larger torque, *M*, increases the trend for printed threads to roll. Moreover, the bioink behind the droplet impart a downward extrusion component, *F*_*e*_, which enhances the sliding tendency of the droplet. The oblate droplets in multi‐DoF printing can effectively avoid the aforementioned issues, whereas a larger contact area with the printing plane can also increase the interface friction, *f*, with the result that the printed threads stick to the plane regardless of the inclination angle, thus enabling all‐direction in situ printing for complex surfaces (Figure 3d). Microscopic photographs of freshly printed droplets support this hypothesis (Figure 3e).

In general, conventional bioprinters are based on a frame structure, with the workspace lying within the frame. In the bioprinting process, defects requiring repair must be placed in the frame, which implies that the bioprinter should be large enough to wrap around the skin wound. Compared to the three‐axis printing platform, the six‐DoF manipulator has a much wider workspace, with the extra DoF providing greater flexibility and effectively avoiding interference between the print head and the printed structure. By utilizing robot‐assisted multi‐DoF bioprinting, it is possible to print a 20‐layer‐thick lattice structure on a vertical plane without the structure collapsing (Movie S2); it is also possible to print on inverted surfaces in cramped corners (Figure 3f,g; Movie S3). To demonstrate the adaptability of this novel skin repair method, we attached freshly collected pigskin to a cube and printed the letters “THU” on three different composite surfaces simultaneously (Figure 3h; Movie S4), thereby further confirming the superiority of this innovative method.

### 3.3 Cell culture and characterization between robotic bioprinting and manual implantation

In the selection of seed cells, adult cells, especially keratinocytes and fibroblasts, were chosen in most researches.^21^, ^25^, ^41^ Amniotic fluid‐derived stem cells (AFSCs) were also a favorable choice in skin repair for their multipotent, as conducted in Skardal's research.^17^ In consideration of the functional activities of regenerated skin tissue, especially necessary appendages reconstruction, chamber assay was used for reference.^42^, ^43^ It is the representative method for reconstructing the hair follicle structure, and has revealed that the reconstitution of appendages from disassociated cells requires epithelial‐mesenchymal interactions.^44^ And several studies have demonstrated the potential of progenitor stem cells to make hair follicles structure in skin substitutes.^45^, ^46^ Based on this, we applied Epi‐SCs and dermal stem cells collected from neonate mouse as epithelial cell source and mesenchymal cell source.

**FIGURE 4:**
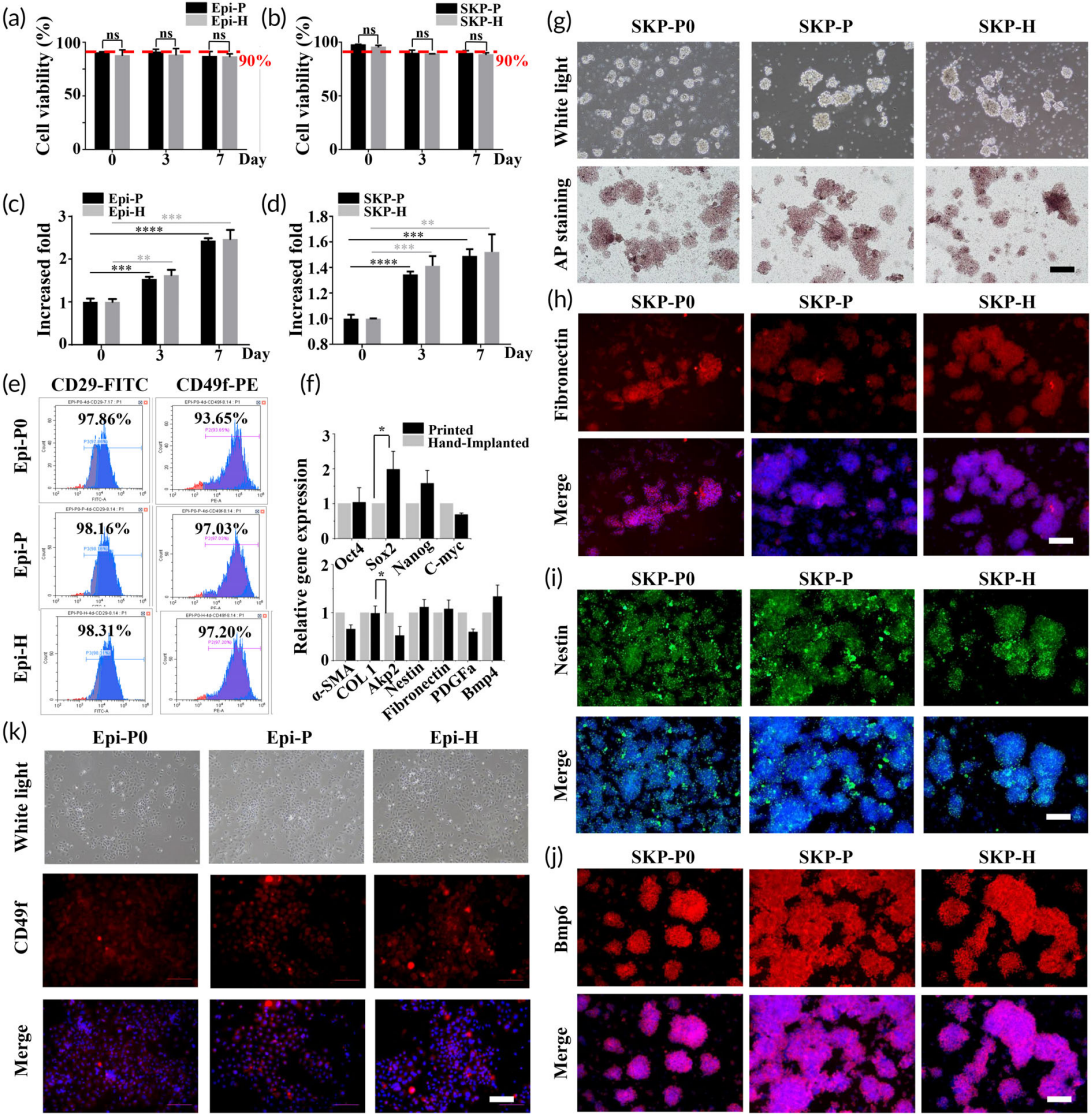
Cell culture and characterization between robotic bioprinting and manual implantation. (a and b) Cell viability of epidermal stem cells (Epi‐SCs) and skin‐derived precursors (SKPs) cultured for 7 days after robotic bioprinting and hand implantation. (c and d) Cell proliferation of Epi‐SCs and SKPs cultures for 7 days after robotic bioprinting and hand implantation. ****p < 0.0001, ***p < 0.001, **p < 0.01. (e) Flow cytometry analysis showed high expression of CD29 and CD49f in Epi‐SCs after normal culture, robotic bioprinting, and hand implantation. (f) Real‐time polymerase chain reaction analysis of SKPs for their expression of genes involved with stemness and hair genesis.* *p* < 0.05. (g) Alkaline phosphatase activities of SKPs in normal culture, robotic bioprinting, and hand implantation. (h–j) Immunofluorescence analysis of fibronectin (red), nestin (green), Bmp6 (red) expression in SKPs in normal culture, robotic bioprinting, and hand implantation. (k) CD49f expression of Epi‐SCs in normal culture, robotic bioprinting and hand implantation (scale bar: 100 μm)

To verify the characterization of the primary stem cells isolated from neonatal mice and to explore the impact of printing process on the cells, the cells were cultured in a Petri dish for 7 days to evaluate cell viability and proliferation after robotic bioprinting and hand implantation. The cells grew well in culture as the viabilities of Epi‐SCs and SKPs were always maintained at approximately 90% in 7 days, and there was no significant difference (*p* > 0.05) between bioprinting and hand implantation (Figure 4a,b). In terms of cell proliferation, the Epi‐SCs grew with adherence to the plate, whereas the SKPs grew in small aggregates and were suspended in the culture. This led to a gradual upward trend in the number of Epi‐SCs over 7 days. SKPs proliferated significantly in the first 3 days, and slowed down in the next few days, which might owe to limitations in the nutrition interchanges caused by aggregation. But both cell types showed significant proliferation during the 7‐day's culture (Figure 4c,d). The diameter of the SKP aggregates was measured and compared between the ordinary culture, printing, and hand implantation environments, revealing that the average diameter of the aggregates was about 80 μm, without significant differences among each method. It has been reported that the expression of alkaline phosphatase (AP) correlates strongly with the occurrence of hair follicles in dermal papilla (DP) cells, and SKPs are similar to DP cells and have a synergistic effect with respect to hair follicle generation.^47^, ^48^ First, we evaluated the influence of bioprinting on the SKPs through the expression of AP, with AP staining revealing that all SKPs exhibited a high expression of AP, with few differences in ordinary culture (SKP‐P0), bioprinting (SKP‐P), and hand implantation (SKP‐H), as shown in Figure 4g. Further characteristic markers of SKPs, namely fibronectin, nestin, and bmp6,^49^ were also detected by immunofluorescent staining, which showed no significant differences between the SKP‐P and SKP‐H cells, but the expression of the three markers increased compared to SKP‐P0 (Figure 4h–j). The Epi‐SCs grew and spread strongly in the Petri dishes without significant morphological differences being observed between the normal culture (Epi‐P0), bioprinting (Epi‐P), and hand implantation (Epi‐H). Moreover, the expression of typical marker CD49f^50^ increased in the printed and hand‐implanted groups compared with the normal culture group (Figure 4k). Flow cytometry confirmed that CD29 and CD49f were expressed strongly and positively in all groups, while the expressions of Epi‐P and Epi‐H were higher than Epi‐P0 (Figure 4e). Stem cells are fragile and sensitive to mechanical disturbance. Maintaining high levels of cell stemness is one of the obstacles to apply stem cells into clinical trials.^51^ To investigate the influence of the bioprinting process on SKP stemness, the pluripotent genes Oct4, Sox2, Nanog, and c‐Myc were detected via real‐time PCR analysis after culturing for 3 days. The results showed that pluripotency was maintained both in the printed and hand‐implanted groups, further implying that our bioprinting robot has little influence on cell stemness (Figure 4k). Additionally, a series of typical genes involved in the hair induction process of SKPs were detected.^47^ The results showed that both the bioprinting and hand‐implanted groups had a high expression of these functional genes, suggesting that the SKPs maintained a high potential to support hair follicle regeneration after the bioprinting process (Figure 4f). These results indicate that these skin‐derived stem cells possess the capacity to achieve in vivo skin repair with appendages, and the proposed bioprinting robot will not impede the functional expression of stem cells.

### 3.4 Validation of the bioprinting process in a murine full‐thickness wound model with de novo hair genesis

At present, there are few studies on the reconstruction of functional skin tissue with hair follicle structure through tissue engineering approach. Representative methods normally reconstitute hair follicles with the help of a physical chamber to confine the epidermal and dermal stem cells suspension to specific areas of skin, or through subcutaneous injection.^52^ Previously, Lee et al. prepared Matrigel chambers through casting and achieved well skin regeneration,^53^ which freed the mice from cumbersome physical chambers and demonstrated the supportive effect of Matrigel on functional skin regeneration. As a complement and further development to this method for precise and automated operation objectives, we introduced Matrigel into 3D bioprinting and used it as a supporting biomaterial of the bioink to provide a favorable microenvironment for cell proliferation and differentiation, as well as desired compressive and tensile strength simulating the native tissue. As revealed in previous studies, Matrigel contains several major ECM proteins and specific amino acid sequences among them provide multiple adhesion sites for the attachment of stem cells and promote differentiation and angiogenesis,^54^ thereby beneficial for the skin‐derived stem cells.

In terms of mechanical properties and printability, the elastic modulus of Matrigel has been proven to be around 450–650 Pa with different measurement,^55^, ^56^ and it is similar to the storage modulus of decellularized skin ECM,^57^, ^58^ which can simulate the growth environment of skin‐derived stem cells. Matrigel undergoes gelation at temperatures in the range 22–37°C and presents the shear‐thinning properties, which are desirable for in situ bioprinting because of the continuous and smooth extrusion.^59^ Besides, the printed structure can be gelled at the skin wound without introducing additional cross‐linking agents. Aimed at this, an extrusion unit equipped with a quick‐response temperature control was integrated into the bioprinting robot to perform subsequent in vivo printing research.

**FIGURE 5:**
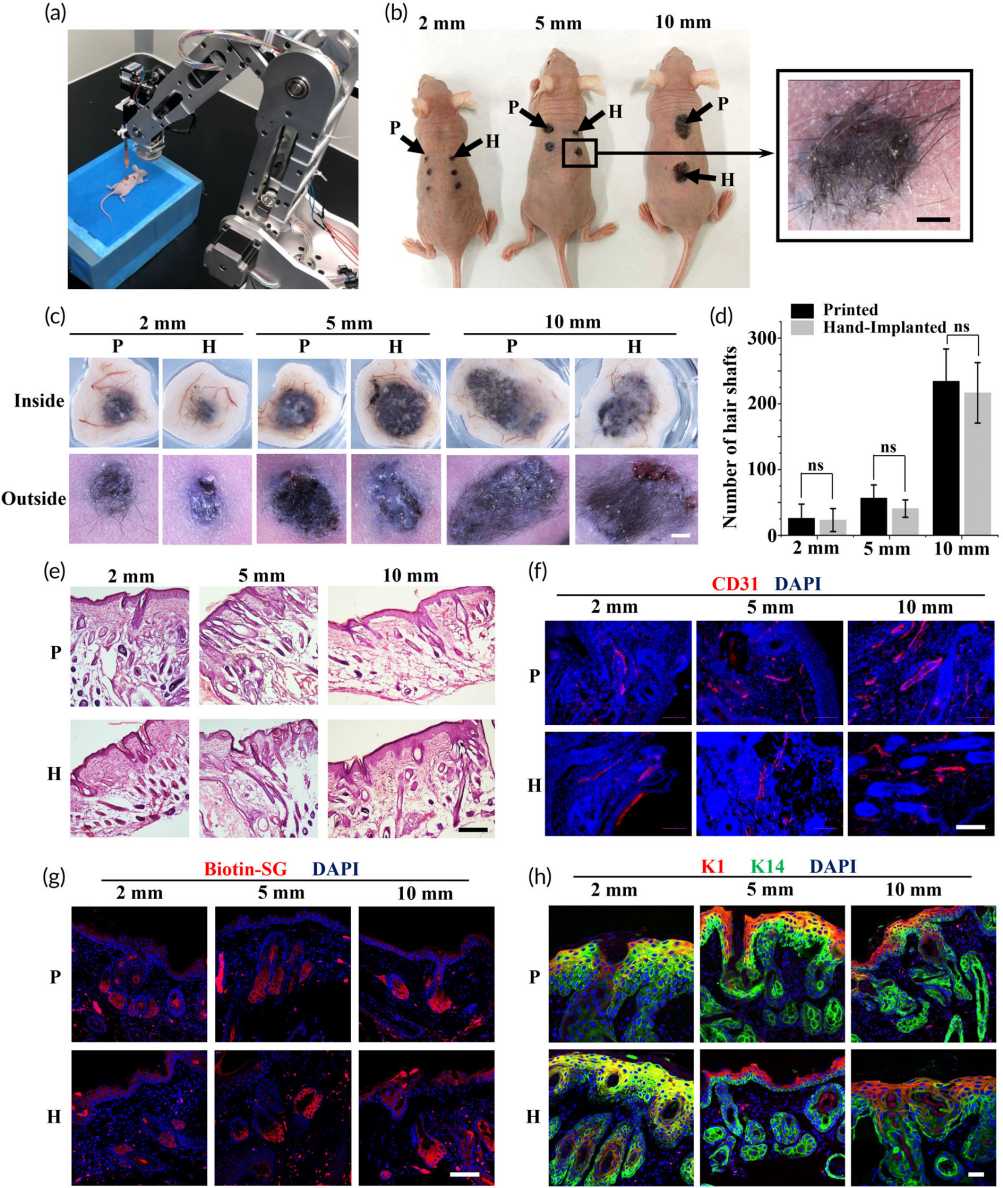
Validation of robotic bioprinting in a murine full‐thickness wound model with de novo hair genesis. (a) Bioprinting robot performs in situ bioprinting on the back of the mouse. (b) Hair follicles generated in 4 weeks after robotic bioprinting and hand implantation (scale bar: 2 mm). (c) Inside and outside of the regenerated skin (scale bar: 2 mm). (d) The numbers of hair shafts per wound in each wound and each group (*n* = 4). (e) H&E staining images of the regenerated skin tissues, which shows epidermis, dermis, and hair follicles (scale bar: 200 μm). (f) Blood vessels in regenerated skin detected through IF staining of CD31 (scale bar: 200 μm). (g) Sebaceous glands detected by IF staining of Biotin (scale bar: 100 μm). (h) Epidermis and dermis detected by IF staining of keratin 1 and keratin 14 (scale bar: 20 μm)

In animal experiment, a murine full‐thickness excisional wound model was used to validate our proposed bioprinting approach for skin repair. Wounds with diameters of 2, 5, and 10 mm were created on the back of BALB/c nu/nu female mice. During the bioprinting process, the robot automatically recognized and tracked skin wounds when unintended motions, for example, breathing and twitching, occurred. The quantitative Epi‐SCs, SKPs, and Matrigel mixture were prepared according to Method Section and loaded to the bioprinting robot. The bioink was in situ printed into the excisional wounds in accordance with the preset path (Figure 5a; Movie S5), with photographs captured before and after bioprinting (Figure S4). In the 4‐weeks' feeding, the mortality rate was 0% and no wound infection of major skin irritation was noted on any mouse. After 4 weeks, the newly regenerated hair‐inclusive skin tissues were photographed for all wounds under a dissecting microscope, and the relevant hair shafts were counted (Figure 5b,d). The average number of hair shafts increased as the wound area increased in the bioprinting group, with the same trend observed in the hand‐implanted group. Bioprinting promoted slightly greater hair follicle growth than observed in the control group, but the difference was not significant (Figure 5f). The regenerated skin tissues underwent H&E staining, revealing densely populated hair follicles and sebaceous glands (Figure 5c), and the latter were further confirmed by immunofluorescence (IF) staining with anti‐Biotin, which identifies sebaceous glands specifically (Figure 5g).^28^ In addition, the presence of newly formed blood vessels in the regenerated skin was confirmed by IF staining with CD31, a marker typically expressed in endothelial cells to detect angiogenesis (Figure 5e).^60^

IF staining for keratin 1 and keratin 14, which are expressed by differentiated keratinocytes and Epi‐SCs^61^, ^62^ showed a stratified epidermis in the regenerated skin of all diameter wounds (Figure 5h). Additionally, the printed skin tissues and native skin integrated well and it's difficult to distinguish the boundary of them under a fluorescence microscope, owing to the growth factors and cytokines in Matrigel that induce cell migration and angiogenesis.^54^ To evaluate the durability of the neogenic hair shafts and the biocompatibility of the bioink, partial printed‐treatment mice were fed for another 10 months after bioprinting for further observation. The results indicated that a substantial proportion of neogenic hair shafts remained after 10 months, with only a few shed. Moreover, H&E staining of the 10‐month regenerated skin tissue indicated that hair follicles remained even after the hair shafts fell out. These results revealed that regenerated skin tissues through our proposed method were permanent in structure and functions, which were close to native healthy skin and showed no signs of teratoma formation (Figure S5). As such, this study demonstrates that the combination of robotic in situ bioprinting and the bioactive bioink presents an effective opportunity for functional skin repair and has a promising clinical translational potential for rapid and accurate skin repair.

## 4 DISCUSSION

At present, there are several 3D printed devices applied to clinical settings with the United Stated Food and Drug Administration's permissions, including surgical implants etc.,^63^ and it reveals the potential of 3D‐printing technology in medical translation. However, these applications of 3D printing have concerned non‐living constructs and there is also an urgent clinical need for 3D bioprinting to provide new paths for regenerative medicine. The translation of bioprinting to medicine have faced significant hurdles, in particular, replicating the 3D structure of tissues precisely at the macro‐scale, simulating the components and possessing corresponding biological functions at the micro‐scale, as well as efficient surgical procedures,^64^, ^65^ which requires targeted exploration and solutions.

Taking skin repair for instance, conventional autologous skin grafts and artificial skin substitutes are not able to satisfy the large‐area skin treatments and personalization requirements for limited sources or rigid fabrication procedure.^10^, ^11^ Complex structure of skin, containing layered epidermis, dermis and important appendages with intricate spatial arrangements, renders the 3D bioprinting of the full skin organ challenging.

Aiming at these challenges, we proposed a novel strategy for skin wound repair to hit the key requirements of prompt cure, personalization and functionality. This method comprises an adaptive multi‐DoF in situ bioprinting robot and functional bioink supporting appendages‐inclusive skin repair. Herein, the concept of bioprinting robot integrating 3D scanning and closed‐loop visual system as well as a manipulator was proposed for the first time to provide rapid on‐site management of full‐thickness wounds. The bioink was deposited quantitatively and precisely avoiding errors caused by irregular wounds and unexpected motions, which achieved accurate skin repair macroscopically. In conventional three‐axis bioprinting process, the nozzle is constrained in the 2D plane along the direction of gravity, making it inevitable to generate stair‐step effect between neighboring layers, which causes surface distortion of the structure.^66^ In addition, curved surface of skin requires the nozzle to adaptively operate in the normal direction according to the specific surface shape, which is insufficient in conventional bioprinter. Under the need of fidelity and the anisotropically growing pattern, our series manipulator can perform stereotactic bioprinting due to the extra‐DoF, which allows the print‐head to adjust the printing direction according to the topography of the skin, making it feasible for skin with complex features, such as large inclination or downward surfaces.

**FIGURE 6:**
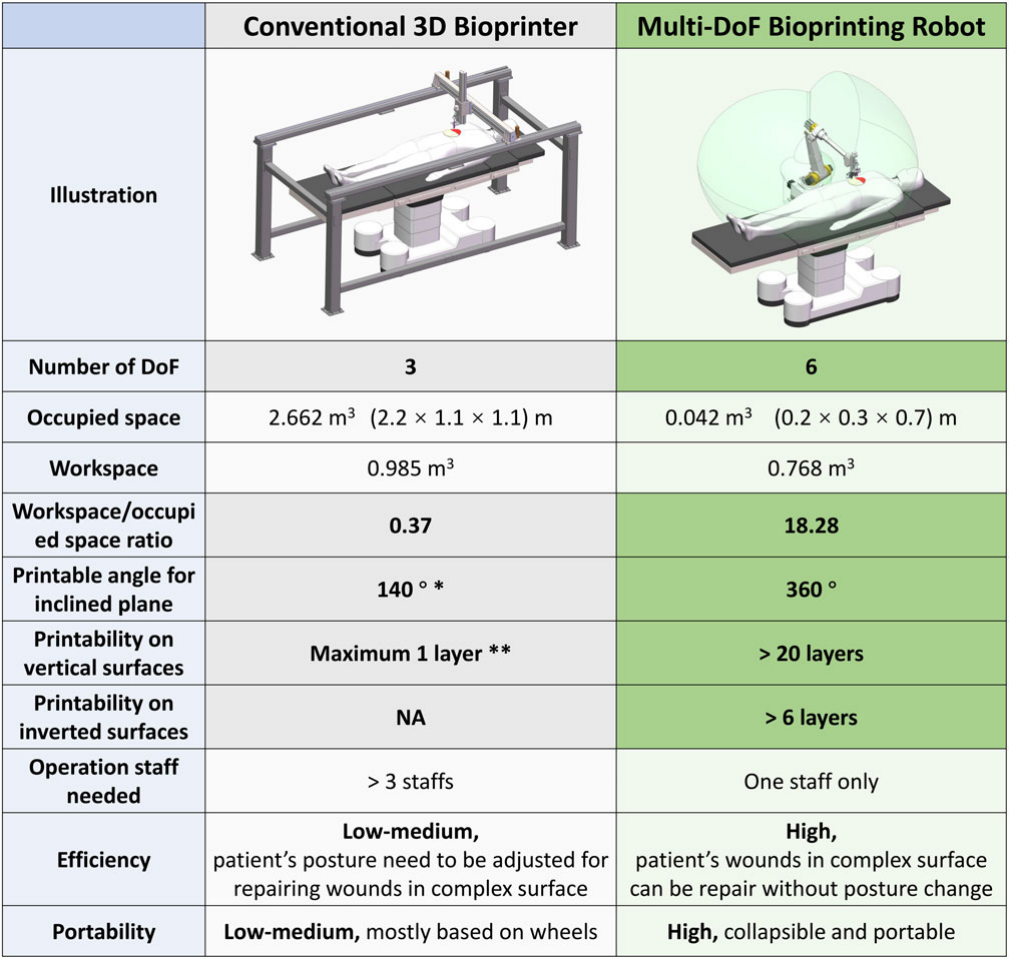
Comparison between conventional bioprinter and robotic bioprinter. *140° is calculated as double 70° in the symmetrical direction. **Maximum 1 layer means that the bioink cloud adheres to the vertical surfaces by increasing the viscosity of the material; however, the print head will still interfere with the printed structure

Furthermore, compared with the traditional three‐axis motion system, the robot provided a larger operation space with a smaller instrumentation volume. Figure 6 represents the illustrations and comparison of the convention 3D bioprinter and robot bioprinter in a clinical setting. In order to make the workspace cover a patient with 1.8 m in height, the occupied space of a three‐axis bioprinter is around 2.66 m^3^, while the robot only takes 0.042 m^3^, which highlights the superiority of the later in portability. Moreover, the use of robot could avoid the risk of contamination. This advantage reflects the potential of the new 3D printing modality for clinical scenarios including X‐ray exposure or pandemics infection (COVID‐19), which can reduce the risk, and better protect the medical staff.

The application of bioprinting techniques to fabricate skin tissue substitutes for wound healing has been explored in previous studies, but rare of them achieved functional skin repair and far away from mature products of skin substitute due to the lack of skin appendages. The replication of the native skin in morphological and physiological properties needs the incorporation of additional cell types and patterning of representative ECM components, and cannot be achieved simply rely on mimic architectural facets of living tissues.^67^

In terms of cell type, as mentioned above, keratinocytes and fibroblasts were mostly used as seed cells for their extensive resources. Different printing processes including inkjet‐based bioprinting,^9^ extrusion‐based bioprinting^23^ and laser‐assisted bioprinting^21^ were applied to print a bilayered skin construct in vitro or directly into the defect site (in situ bioprinting). Most results showed high viabilities of printed cells after bioprinting as well as collagen secretion of fibroblasts and K14 expression of keratinocytes. While the drawback was also prominent that little changes were shown in subsequent culture due to the low secretion activity and proliferation rate, which might be the main obstacle for further clinical application. High proliferation rate and functional activities of seed cells may be the complementary key to tissue construction and regeneration in vivo, which makes stem cells a favorable choice, including induced pluripotent stem cells (iPSCs),^68^ mesenchymal stem cells,^24^ and AFSCs. Though favorable wound closure has been achieved in these studies, the essential appendages in skin were still missing, and it inspired us to search for ideas from the research of hair follicle construction.

Previous studies involving hair follicle construction demonstrate that there are multipotent stem cells with the capacity to regenerate hair follicles and sebaceous glands in adult mammalian skin and this multipotency can be maintained in cell culture.^69^ The hair‐differentiation potentiality of Epi‐SCs can be activated by SKPs, and co‐grafting of those cellular components from mice allows complete hair reconstitution,^70^ which has also been proved in our previous study.^71^ Compared with the cells used in most existing studies, these primary stem cells are promising for seed cells in 3D bioprinting skin constructs for their good proliferation, biological activities as well as the capacity of directional differentiation into fully functional skin.

However, most of de novo hair regeneration was observed in rodents, while demonstrating these regenerative properties in human cells and tissues has been challenging for human DP cells immediately lose their hair inductive potential after in vitro culture. Jahoda et al.^72^ proposed that the striking difference between the behavior of human DP cells and those of mice is their propensity and capacity to aggregate both in vitro and in vivo. Based on this, Higgins et al.^73^ created a 3D microenvironment to help human DP cells self‐aggregation, which represented a significant advance in using cell‐based therapy for hair‐follicle neogenesis. In addition, there are also studies built biomimetic microenvironments to improve human hair follicles regeneration in recent years,^74^, ^75^ which inspired us for subsequent research. The difference between human DP cells and rodent DP cells is one of the major challenges hindering the translation of skin repair research to clinic, and it requires broader concerns.

Biomaterials, the component of tissue scaffolds, is another focus in skin 3D bioprinting. Naturally derived materials are the most popular biomaterials in skin 3D bioprinting. Type I collagen is one of the main components in the ECM of dermis secreted by fibroblasts, making it the first choice for most researches.^17^, ^21^, ^25^ In addition, fibrinogen, alginate, and polyethylene glycol have also been applied to improve printability and mechanical properties.^9^, ^23^ But for skin‐derived stem cells, the compatibility of these materials has not been verified. The drawbacks might be the inappropriate mechanical properties, potential risks brought about by the crosslinking agents including glutaraldehyde or calcium chloride, as well as the absence of adhesion sites for cells, which go against the differentiation and functional expression of stem cells. The composition of Matrigel is similar to ECM for its basement membrane‐like nature, thus it was selected as the biomaterial in this research. Matthias et al. noted that Matrigel has advantages over artificial biomaterials in directing stem‐cell rate.^76^ The growth support of Matrigel on DP and sweat glands has also been verified in previous study.^77^, ^78^ The interaction of skin‐derived stem cells with Matrigel acts in concert to give rise to a series of spatially and temporally coordinated events that regulate the stem‐cells fate, specifically, directional differentiation into epidermis and dermis with necessary appendages.

Although the cytocompatibility of Matrigel is favorable, the clinical promise is limited due to its tumor‐derived, ill‐defined and variable composition.^79^ The potential for antigenicity is one of the inherent limitations. As it is animal‐derived, Matrigel might contain xenogenic contaminants, and the presence of growth factors and other biological proteins can lead to undesirable cellular effects, which limits its further application in other animal studies and even clinical trials. In addition, the mechanical properties of Matrigel show variability between batches, and it leads to an uncontrollable behavior when standard pneumatic‐driven dispensing system applied, which needs additional procedures to regulate the printing process, such as volumetric‐based extrusion and quick‐response temperature control.^80^ Even so, Matrigel can only provide the ECM‐similar mechanical properties but cannot immediately present the apparent strength of skin tissues including tensile and compression modulus in kilopascal scale.^81^ Thus, there might be potential risks for impairment in the structural integrity at the initial stage after bioprinting due to the scratching of mice, which also requires further improvement in the future. Herein, the Matrigel was used mainly for its favorable support for cell proliferation and migration. For future clinical translation of this strategy, synthetic biomaterials with highly biocompatibility, stable mechanical and apathogenic properties should be developed to provide appropriate alternatives to Matrigel.

This research reports the development of an adaptive bioprinting robot, based on which, we performed a direct location‐specific printing with Epi‐SCs, SKPs extracted from neonatal mice and Matrigel as bioink on a murine full‐thickness excisional wound model. The evaluation of printability, efficiency, and therapeutic effect indicated that this method is a promising strategy for skin wound repair with the potential for clinical translation. But before that, these aspects need more attention and to be further studied: (i) verifying the advantages of the bioprinting robot over hand‐implanted method on large animal models, such as porcine full‐thickness excisional wound, which is what we are currently focusing on; (ii) developing alternative synthetic biomaterials to Matrigel with tunable mechanical and apathogenic properties while maintaining biocompatibility for wider application; (iii) exploring solutions of human‐derived skin cells expressing insufficient hair regeneration potentials in vitro; (iv) searching for alternative seed cells in skin bioprinting. For instance, recent studies proved that both epithelial stem cells and skin‐derived precursor cells can be induced from iPSCs,^82^, ^83^ and these results suggested an approach for generating a large number of Epi‐SCs and SKPs for tissue engineering for wound healing without sacrificing neonatal mice.

## 5 CONCLUSION

This study describes the proof‐of‐concept demonstration of an adaptive bioprinting robot to provide rapid on‐site bioprinting using Epi‐SCs, SKPs, and Matrigel as bioink on a murine full‐thickness excisional wound model. The 3D scanning and closed‐loop visual system integrated in the robot and the multi‐DoF mechanism provide immediate, precise, and complete wound coverage through stereotactic bioprinting, which are important for maintaining homeostasis, wound closure, epithelialization, and scar prevention. Matrigel provides a biomimetic ECM that serves as structural support for skin‐derived stem cells, and stimulates the latter to differentiate into mature skin tissue that closely matches native skin, complete with epidermis, dermis, blood vessels, hair follicles and sebaceous glands etc. This study provides an effective strategy for skin repair through the combination of the novel robot and a bioactive bioink, and has a promising clinical translational potential for further applications.

## CONFLICT OF INTEREST

The authors declare that they have no conflict of interest.

## AUTHOR CONTRIBUTIONS

**Wenxiang Zhao:** Conceptualization (lead); data curation (lead); formal analysis (lead); investigation (lead); methodology (equal); project administration (lead); software (lead); validation (equal); visualization (equal); writing – original draft (lead); writing – review and editing (lead). **Haiyan Chen:** Conceptualization (equal); data curation (equal); investigation (equal); methodology (equal); project administration (equal); resources (equal); validation (equal); visualization (equal); writing – original draft (equal); writing – review and editing (equal). **Yi Zhang:** Data curation (equal); methodology (equal); validation (equal); writing – original draft (equal). **Dezhi Zhou:** Data curation (supporting); methodology (supporting); validation (supporting). **Lun Liang:** Methodology (equal); resources (supporting). **Boxun Liu:** Data curation (supporting); methodology (supporting); validation (supporting). **Tao Xu:** Conceptualization (equal); project administration (equal); resources (equal); supervision (equal).

### PEER REVIEW

The peer review history for this article is available at https://publons.com/publon/10.1002/btm2.10303.

## Supporting information


**Appendix S1**: Supporting Information

**Movie S1** Structured light scanning of the skin wounds in the back of mouse.

**Movie S2** A 20‐layer lattice structure printed on vertical surface by the robotic bioprinter.

**Movie S3** A 6‐layer lattice structure printed on inverted surface in cramped corner by the robotic bioprinter.

**Movie S4** The letters “THU” printed on three different composite surfaces by the robotic bioprinter.

**Movie S5** In situ bioprinting process performed on the back of mouse.

## Data Availability

The data that support the findings of this study are available from the corresponding author upon reasonable request.
