# Supplementary material for: Adaptive multi‐degree‐of‐freedom in situ bioprinting robot for hair‐follicle‐inclusive skin repair: A preliminary study conducted in mice
Source: Bioeng Transl Med. 2022 Feb 28;7(3):e10303. doi: 10.1002/btm2.10303 (PMC9472011; doi:10.1002/btm2.10303)
Supplement: Supplementary file 1 — Appendix S1: Supporting Information [file BTM2-7-e10303-s001.docx]

Supporting Information

Adaptive multi-degree-of-freedom in situ bioprinting robot for hair-follicle-inclusive skin repair: a preliminary study conducted in mice.

Wenxiang Zhao, Haiyan Chen, Yi Zhang, Dezhi Zhou, Lun Liang, Boxun Liu, Tao Xu^*^

This file includes: Figure. S1 to S5, Tables S1 and Movies S1 to S5.


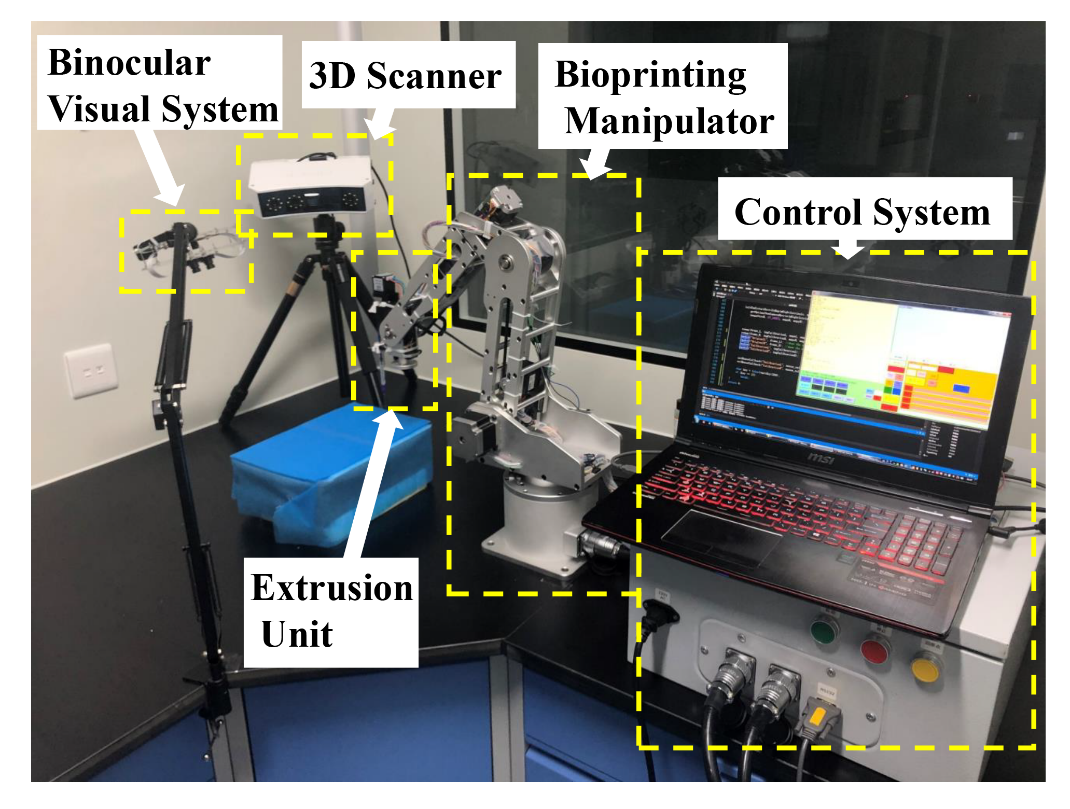


**Figure. S1.** The composition of the adaptive multi-degree-of-freedom *in situ* bioprinting robot.


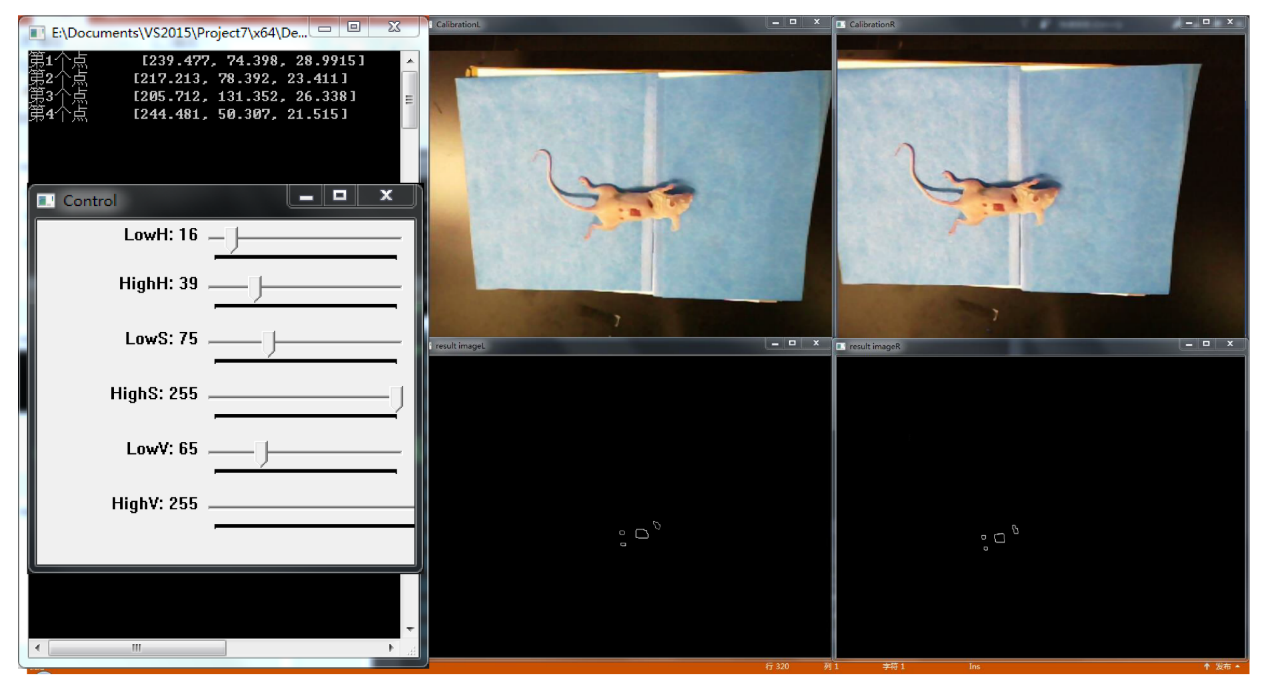


**Figure. S2.** The main interface of the binocular visual recognition system.


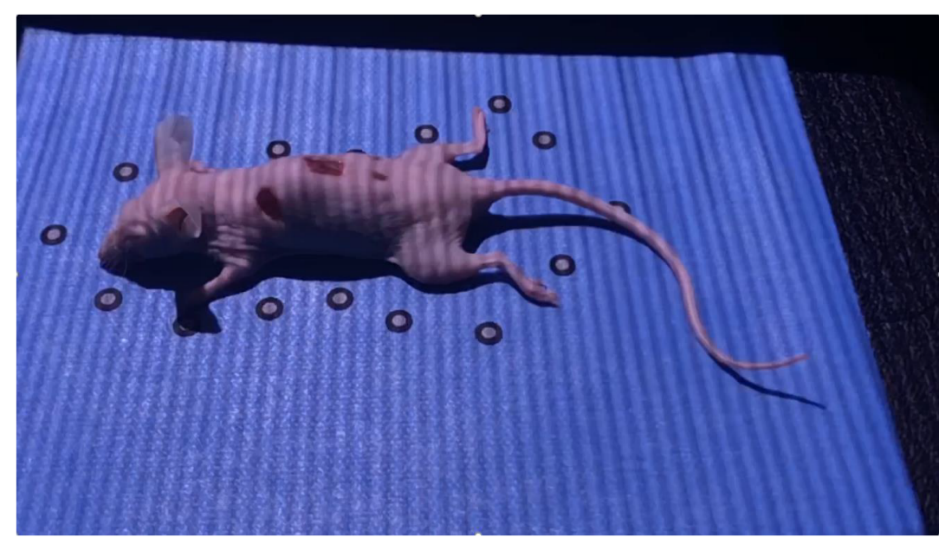


**Figure. S3.** Structured light scanning of the skin wounds on the back of mouse.


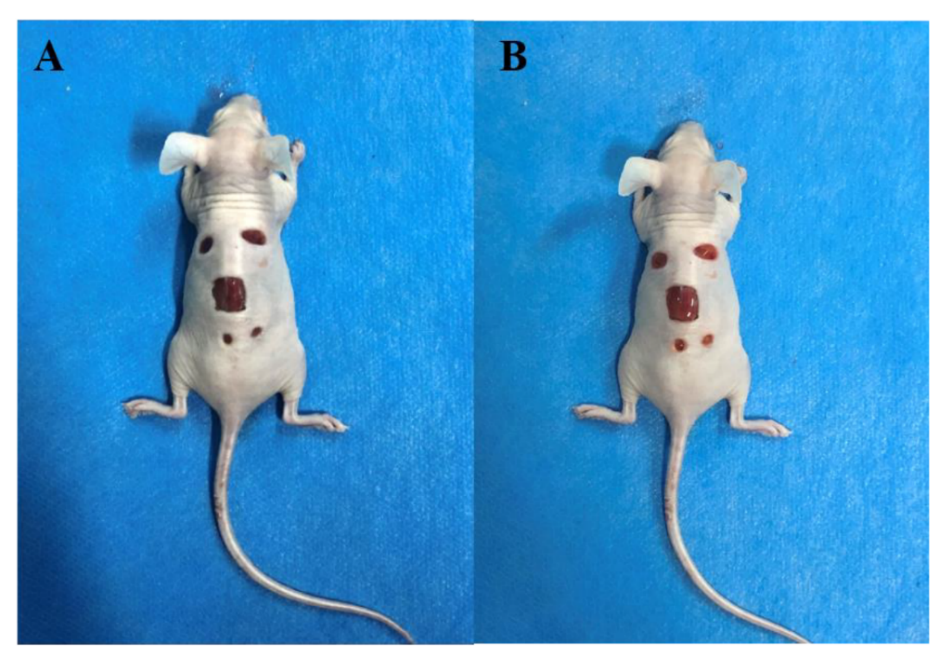


**Figure. S4.** Before (A) and after (B) *in situ* bioprinting on the back of mouse for skin wounds with various shapes.


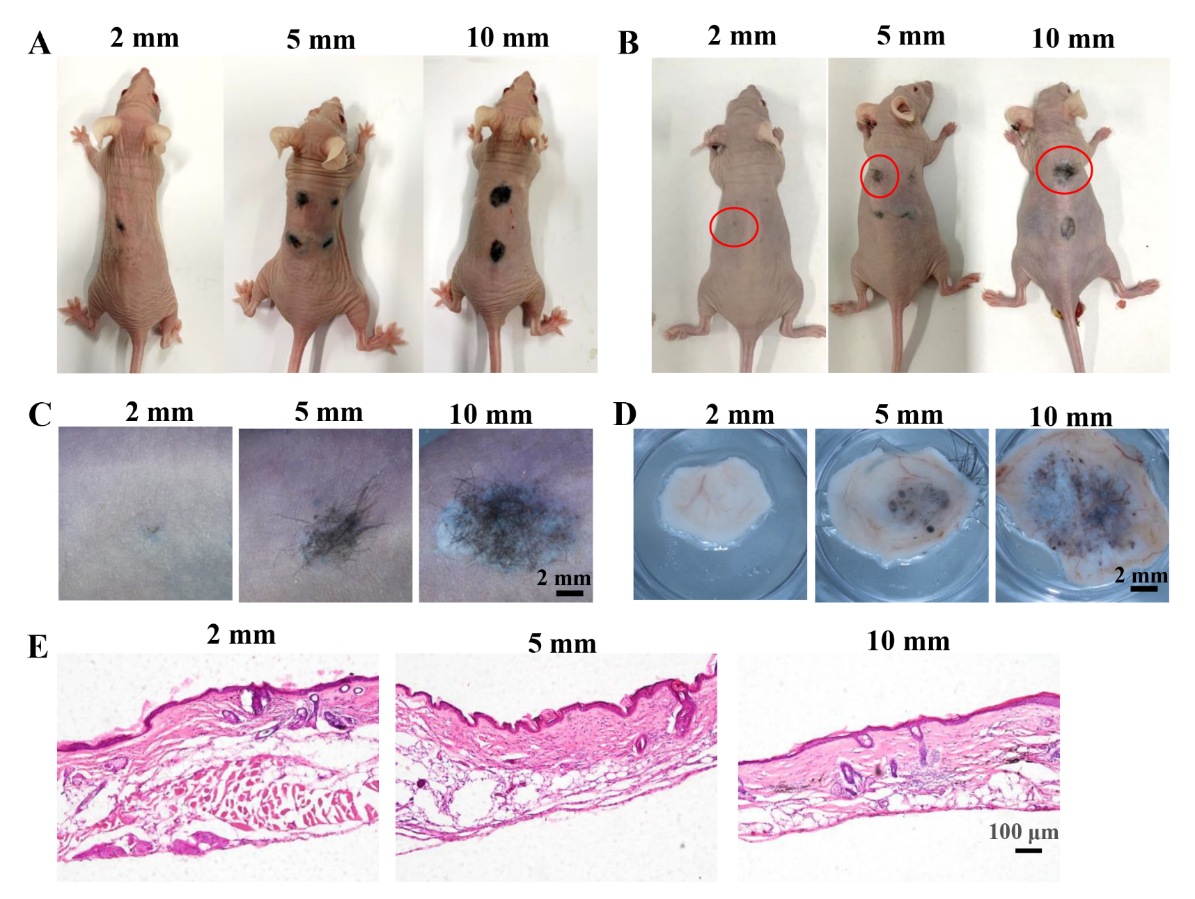


**Figure. S5. Evaluation of biocompatibility and stability.** (A) Representative images of regenerated skin with hair shafts in 4 weeks after robotic bioprinting. (B) Representative images of regenerated skin with hair shafts in 10 months after robotic bioprinting, denoting part of the hair shafts fell out. (C, D) The regenerated skin section of the mice kept for 10 months was picked and the outside and inside were captured by dissecting microscope. (E) H&E staining analysis showed there was no teratoma formation in regenerated skin after 10 months and the skin is close to healthy native skin with hair follicles, epidermis, dermis etc.

| **Item** | ***θ_i_* (°)** | ***d_i_*(mm)** | ***a_i_*(mm)** | ***α_i_*(°)** |
| --- | --- | --- | --- | --- |
| ***i*=1** | *θ*_1_ | 203 | 80 | 90 |
| ***i*=2** | *θ*_2_ | 0 | 260 | 0 |
| ***i*=3** | *θ*_3_ | 0 | 80 | 90 |
| ***i*=4** | *θ*_4_ | 248 | 0 | 90 |
| ***i*=5** | *θ*_5_ | 0 | 0 | 90 |
| ***i*=6** | *θ*_6_ | 64.5 | 0 | 0 |

**Table S1.** The link parameters of the robot

**Movie S1.** Structured light scanning of the skin wounds in the back of mouse.

**Movie S2.** A 20-layer lattice structure printed on vertical surface by the robotic bioprinter.

**Movie S3.** A 6-layer lattice structure printed on inverted surface in cramped corner by the robotic bioprinter.

**Movie S4.** The letters “THU” printed on three different composite surfaces by the robotic bioprinter.

**Movie S5.** *In situ* bioprinting process performed on the back of mouse.
